# Proteome differences of dental stem cells between permanent and deciduous teeth by data-independent acquisition proteomics

**DOI:** 10.1515/biol-2022-0998

**Published:** 2025-01-29

**Authors:** Suping Zhang, Yuqing Liu, Jin Dong, Min Jiao, Yongchun Gu, Liling Chen, Na Yuan, Jianrong Wang, Dezhao Yang, Fanwen Meng

**Affiliations:** Infectious Disease Prevention and Control Department, Suzhou Center for Disease Control and Prevention, Suzhou 215131, China; Cyrus Tang Medical Institute, Soochow University School of Medicine, Suzhou 215123, China; Oral Implantology Department, Suzhou Stomatological Hospital, Suzhou 215005, China; Respiratory Disease Research Institute, Suzhou First Hospital affiliated to Soochow University, Suzhou 215003, China; Department of Stomatology, Suzhou Ninth Hospital affiliated to Soochow University, Suzhou 215200, China

**Keywords:** human dental pulp stem cells, DIA proteomics, protein profile, DPSC, SHED

## Abstract

Dental pulp stem cells hold significant prospects for tooth regeneration and repair. However, a comprehensive understanding of the molecular differences between dental pulp stem cells (DPSC, from permanent teeth) and stem cells from human exfoliated deciduous teeth (SHED, from deciduous teeth) remains elusive, which is crucial for optimizing their therapeutic potential. To address this gap, we employed a novel data-independent acquisition (DIA) proteomics approach to compare the protein expression profiles of DPSC and SHED. Based on nano-LC-MS/MS DIA proteomics, we identified over 7,000 proteins in both cell types. By comparing their expression levels, 209 differentially expressed proteins were identified. Subsequent Gene Ontology and Kyoto Encyclopedia of Genes and Genomes enrichment analyses, along with protein–protein interaction network construction, revealed significant metabolic differences and key regulatory nodes. DPSC exhibited significantly higher expression of proteins belonging to the NDUFB family, SMARC family, RPTOR and TLR3. These proteins are known to be involved in critical cellular processes such as mitochondrial energy metabolism, mTOR-related autophagy pathway, and innate immune response. Conversely, SHED displayed elevated expression of AKR1B family, which participated in glycerolipid metabolism and adipogenic differentiation, PRKG1, MGLL and UQCRB proteins associated with thermogenesis. These findings highlight the specific proteomic landscape of DPSC and SHED, suggesting their distinct biological roles and potential applications.

## 1 Introduction

Dental pulp stem cells (DPSC) and stem cells from human exfoliated deciduous teeth (SHED) represent the two primary subtypes of tooth-derived pulp stem cells [1,2]. Both cell types originate from the dental pulp, the soft tissue within the tooth, distinguishing them from periodontal stem cells such as periodontal ligament stem cells (PDLSC), gingival mesenchymal stem cells (GMSC), and stem cells from apical papilla (SCAP), which are isolated from the surrounding periodontal tissues [3,4]. Since their discovery and characterization, these dental-derived mesenchymal stem cells have attracted significant research interest due to their inherent mesenchymal stem cell properties, including self-renewal, multi-differentiation potential, and low immunogenicity [5,6]. These properties make them highly promising candidates for applications in regenerative medicine and tissue engineering [7,8].

However, due to the distinct origin in terms of host ages and tooth types (permanent and deciduous), DPSC and SHED exhibit significant differences in their biological characteristics and clinical applications. Research indicated that SHED possessed a higher concentration of anti-aging signals, suggesting a potential for enhanced rejuvenation of aging cells most due to their origin from youth tissue [9,10]. Comparative studies have revealed disparities in the proliferation rate, differentiation capability, and cytokine expression profiles between DPSC and SHED. It showed that SHED demonstrated a higher proliferation rate and differentiation capacity *in vitro*, along with superior mineralization potential compared to DPSC [11]. Moreover, SHED exhibited a more pronounced expression of cytokines associated with immunomodulation, odontogenesis, and osteogenesis, while DPSC demonstrated elevated expression of angiogenesis-related cytokines [12] and exhibited significant potential in bone tissue engineering and nerve tissue repairing [13,14].

Despite these intriguing findings, a comprehensive understanding of the protein molecular basis underlying the functional differences between DPSC and SHED remains elusive. Proteins, as the ultimate products of gene expression, are fundamental to cellular function. They are integral to a wide range of essential cellular processes, including metabolic catalysis, signal transduction, substance transport, immune defense, and cell division. Understanding the proteomic landscape of these two stem cell types is critical for optimizing their therapeutic applications. While DPSC and SHED share similar biological characteristics, their differences at the protein level may impact their efficacy and safety in specific applications.

In this study, we aim to conduct a comparative proteomic analysis of DPSC and SHED utilizing the innovative data-independent acquisition (DIA) proteomics technology [15]. By dissecting the protein profiles of these two stem cell populations, we aim to uncover the protein molecular basis for their distinct biological properties and guide future research into their therapeutic applications in tooth regenerative and tissue engineering. This investigation will shed light on the protein molecular basis of their functional differences and provide valuable insights for optimizing their clinical applications.

## 2 Materials and methods

### 2.1 Subjects and cell culture

This study involved the isolation and *in vitro* culture of DPSC and SHED. DPSC was harvested from impacted third molars or other extracted teeth of healthy human subjects aged 16–29 years. SHED was obtained from exfoliated deciduous teeth of children aged 5–8 years. Informed consent was obtained from all participants or their guardians prior to the collection of any samples. Teeth were collected from Suzhou Stomatological Hospital. All experiments were conducted at Soochow University, adhering to all relevant national regulations, institutional policies, and the tenets of the Helsinki Declaration. This research involving human subjects received ethical approval from the ethics committees of Suzhou Stomatological Hospital and Soochow University (Approval Number: SUDA20230727H08).

The tooth samples were placed in PBS buffer after extraction and sent to the laboratory for processing within 12 h. Following the extraction of pulp tissue, primary dental stem cells were cultured using either an adherent culture method or enzymatic digestion with collagenase I (3 mg/mL)/dispase (4 mg/mL) (Sigma, USA) as described previously [16]. Once the cells had grown into fibroblasts, they were subcultured in α-modification of Eagle’s medium (α-MEM, HyClone, USA) supplemented with 15% fetal bovine serum (Gibco, USA), 100 U/mL penicillin‒streptomycin (Beyotime, China), 100 mM l-ascorbic acid phosphate (Sigma, USA), and 2 mM l-glutamine (Gibco, USA). Cultures were maintained in a humidified incubator at 37°C with 5% CO_2_.


**Informed consent:** Informed consent has been obtained from all individuals included in this study.
**Ethical approval:** The research related to human use has been complied with all the relevant national regulations, institutional policies and in accordance with the tenets of the Helsinki Declaration, and has been approved by the Ethics Committees of Suzhou Stomatological Hospital and Soochow University (Approval Number: SUDA20230727H08).

### 2.2 Cell proliferation and identification

Cell proliferation was assessed using a cell counting method. Briefly, 2 × 10^4^ cells were seeded into individual wells of six-well plates and counted at Days 1, 2, 3, and 4. Cell numbers were recorded at each time point. The cell proliferation rate was calculated based on the cell number of the seeded time. Cell proliferation rate = (No_1–4_ − 20,000)/20,000 × 100%.

Dental stem cell identification was performed via flow cytometry (BD Calibur, USA) based on surface marker expression. Cells were collected and stained with antibodies targeting CD29, CD44, CD73, CD90, CD105, CD146, and CD166 (BioLegend, USA). The positive expression rate was analyzed using flow cytometry. Additionally, CD34 and CD45 staining was conducted to exclude potential hematopoietic cell. Data were presented as mean ± SD and statistically analyzed using GraphPad Prism 8.0.

### 2.3 Protein sample preparation and DIA proteome detection

This study employed next-generation label-free DIA quantitative proteomics technology. Six cell samples, including three DPSCs and three SHEDs from different individuals, were selected for DIA proteomic analysis. Cells were collected at passage 3, with a minimum of 5 × 10^6^ cells required for protein extraction. For protein sample preparation, the cell pellets were suspended in cell lysis buffer containing sodium dodecyl sulfate (SDS) and 1× cocktail (with EDTA), followed by ultrasonic dissociation. The lysate was then centrifuged at 25,000*g* for 15 min at 4°C. The supernatant was incubated with 10 mM dithiothreitol at 37°C for 30 min, followed by the addition of 55 mM iodoacetamide and incubation in the dark for 45 min. Five volumes of pre-cooled acetone were added to the lysate and incubated at −20°C for 2 h. After centrifugation at 25,000*g* for 15 min at 4°C, the supernatant was discarded. The protein precipitate was resuspended in an appropriate amount of protein lysate without SDS and sonicated. The final protein sample was obtained after centrifugation at 25,000*g* for 15 min at 4°C.

Following tryptic digestion and high pH reversed-phase separation, the peptide samples were analyzed by nano-liquid chromatography coupled with tandem mass spectrometry (nano-LC‒MS/MS) for data-dependent acquisition (DDA) library construction and DIA quantitative detection. This process was outsourced and completed at BGI, China. For DDA library construction, a pooled sample containing an equal amount of all six samples was subjected to LC–MS/MS for DDA library construction. For DIA analysis, each individual sample was separated using a 120 min high-performance liquid chromatography gradient prior to MS analysis. The gradient was as follows: 0–5 min, 5% mobile phase B (98% acetonitrile, 0.1% formic acid); 5–90 min, linear increase of mobile phase B from 5 to 25%; 90–100 min, increase of mobile phase B from 25 to 35%; 100–105 min, increase of mobile phase B from 35 to 80%; 105–115 min, 80% mobile phase B; 115–120 min, 5% mobile phase B. The LC-separated peptides were ionized by nano-electrospray ionization and analyzed using a Q-Exactive HF tandem mass spectrometer (Thermo Fisher Scientific, San Jose, CA) in DIA mode. The main MS settings were ion source voltage 1.9 kV, MS scan range 400–1,250 *m*/*z*, MS resolution 120,000, and maximum injection time (MIT) 50 ms. The MS scan range was divided into 45 continuous windows for DIA–MS/MS analysis. MS/MS fragmentation was performed using higher-energy collision dissociation with automatic MIT. Fragment ions were detected in the Orbitrap with a resolution of 30,000. The collision energy was set to a distributed mode of 22.5, 25, and 27.5, with an automatic gain control target of 1E6.

### 2.4 Data analysis and differential protein identification

The data analysis consisted of three primary stages: spectral library construction from DDA data, large-scale DIA data acquisition, and subsequent data analysis. Spectral library construction was achieved using DDA data identified by the Andromeda search engine within MaxQuant. These identified results served as the foundation for constructing a comprehensive spectral library. For large-scale DIA data, mProphet algorithm was applied for analytical quality control, ensuring the reliability of the vast quantitative data generated.

MSstats version 2.0 software package was used for quantitative and differential protein analysis [17]. The selection of the protein database is crucial for accurate protein identification. In this study, we chose the non-redundant proteome database, RefSeq, obtained from the National Center for Biotechnology Information. The DIA data were further refined through retention time calibration using iRT peptides. To mitigate false positives, a target-decoy model, commonly used in SWATH-MS analysis, was implemented, achieving a 1% false discovery rate (FDR). This stringent filtering resulted in highly significant quantitative findings. These data were then preprocessed based on the defined comparison groups (SHED vs DPSC). Subsequent statistical significance testing, conducted using a specific model, identified differentially expressed proteins (DEPs) between DPSC and SHED. A stringent filtering process was applied, utilizing a fold change threshold of greater than 2 and a *p*-value less than 0.05, to obtain statistically significant DEPs.

Hierarchical clustering was employed to group these DEPs, providing a visual representation of DEP expression at the sample level. Additionally, enrichment analysis was conducted on the identified DEPs to further explore their biological significance. Figure S1 displays the process of DIA proteomics.

### 2.5 Gene ontology (GO) and Kyoto Encyclopedia of Genes and Genomes (KEGG) pathway enrichment analysis

To delve into the functional implications of the identified DEPs, we conducted GO and KEGG pathway enrichment analyses. The GO analysis, facilitated by the GO plot package within the MSstats software, aimed to elucidate the enrichment of GO terms related to cellular components (CC), biological processes, and molecular functions associated with the differential protein expression.

Furthermore, KEGG pathway enrichment analysis was performed to identify significantly over-represented pathways in the dataset. The classification and bubble chart visualization of KEGG pathways provided a comprehensive overview of pathway enrichment. The Phyper function within R software was utilized for enrichment analysis, calculating the *p*-value for each pathway. Subsequently, the *p*-value was corrected for multiple comparisons using the FDR to obtain the *Q*-value. A *Q*-value threshold of 0.05 was employed to define statistically significant enrichment.

### 2.6 Protein–protein interaction (PPI) network analysis

Proteins often interact with each other to form functional complexes and execute cellular processes. To investigate these interactions, we conducted PPI network analysis by comparing our dataset with the STRING database. This analysis resulted in a network interaction diagram depicting the relationships between DEPs, focusing on the top 100 interactions with the highest confidence levels. This combined approach, encompassing GO and KEGG pathway enrichment analysis alongside PPI network analysis, provided a multifaceted understanding of the functional consequences of differential protein expression between SHED and DPSC groups.

## 3 Results

### 3.1 Primary stem cell culture and identification

**Figure 1:**
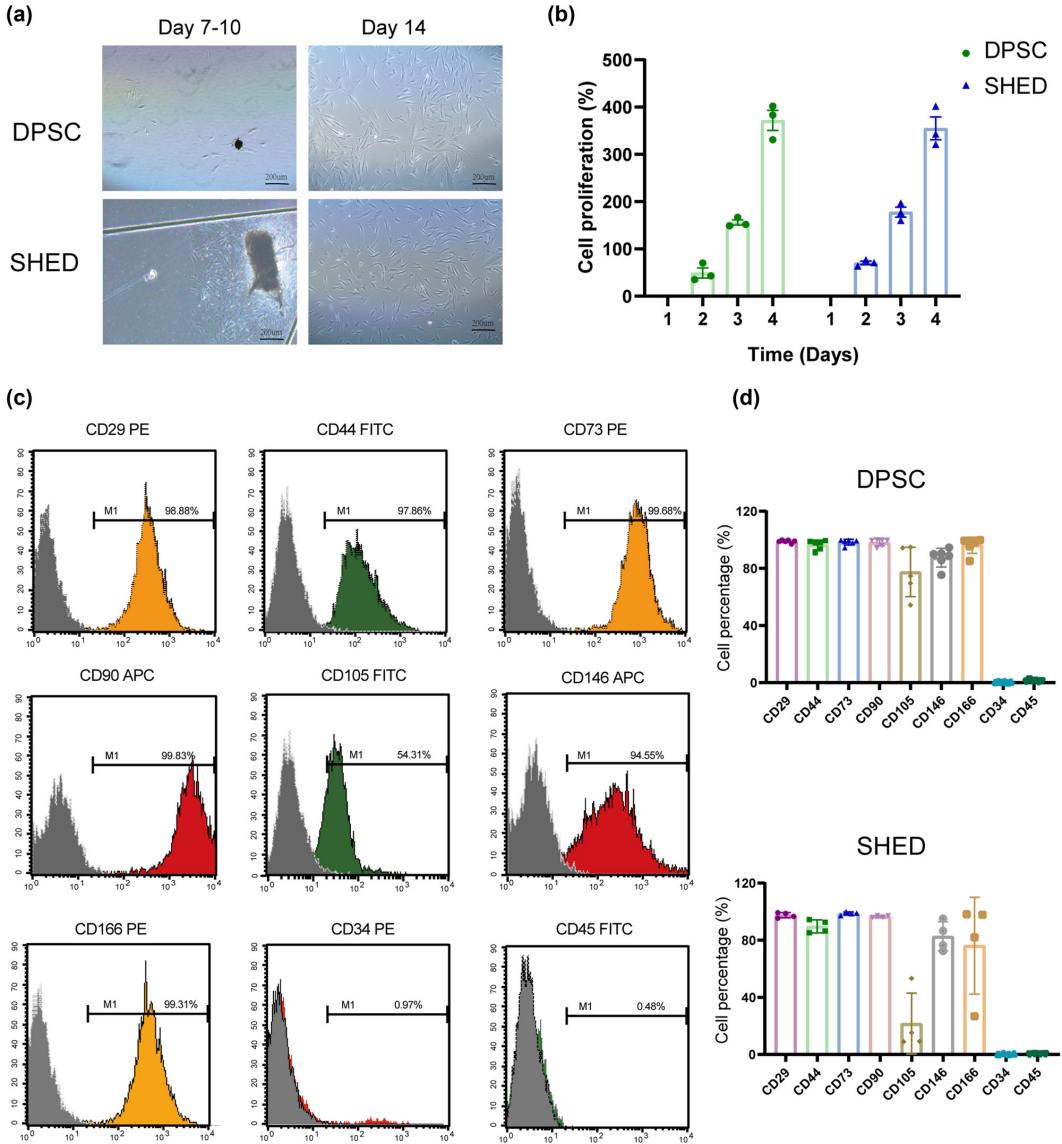
Cell morphology, proliferation, and identification. (a) Microscopic morphology of the cells. (b) Cell proliferation of the cells in different time points. (c) Representative FCM chart illustrating surface marker expression on the cells. (d) Statistical histogram of the expression of markers in DPSC and SHED.

Primary stem cells were isolated using a combined approach of enzymatic digestion and tissue adherence. Cells migrated out of the tissue explants within 7–10 days of culture and were subsequently sub-cultured into 60 mm dishes after 14–20 days. Figure 1a demonstrates the morphology of the cultured cells. Once cells reached confluence, they exhibited a relatively uniform, spindle-shaped, fibroblast-like morphology and displayed characteristic colony formation. Cell proliferation analysis revealed no significant differences in growth rate between DPSC and SHED (Figure 1b), which might be related to the small sample size.

To confirm the stem cell nature of the cultured cells, surface marker expression was assessed by flow cytometry. Both DPSC and SHED exhibited high expression of mesenchymal stem cell markers, including CD29, CD44, CD73, CD90, CD105, CD146, and CD166, while lacking expression of hematopoietic markers CD34 and CD45 (Figure 1c and d). These findings indicate that the cultured cells were indeed dental-derived mesenchymal stem cells.

### 3.2 Differential protein analysis and GO annotation reveals the subcellular protein localization

Following successful culture of primary dental pulp tissue into stable cell lines, a comparative proteomics study using DIA was performed on six individual DPSC and SHED cell lines (three replicates per group). A total of 8,509 proteins were identified across all cells, with 7,662 proteins exhibiting comparable expression levels between DPSC and SHED (data uploaded in public database iProX: IPX0009667000). Database and software analysis revealed an average of 7,114 ± 184 proteins in DPSC and 7,136 ± 138 proteins in SHED. Figure S2 shows the CV value and PCA analysis of DIA quality control between DPSC and SHED. For clinical human samples, due to the individual differences between samples, the experiment is considered stable and reliable when the median CV value is below 30%. In this study, the median CV values of DPSC and SHED groups were between 15 and 25%, indicating that the experiment was stable and reliable.

**Figure 2:**
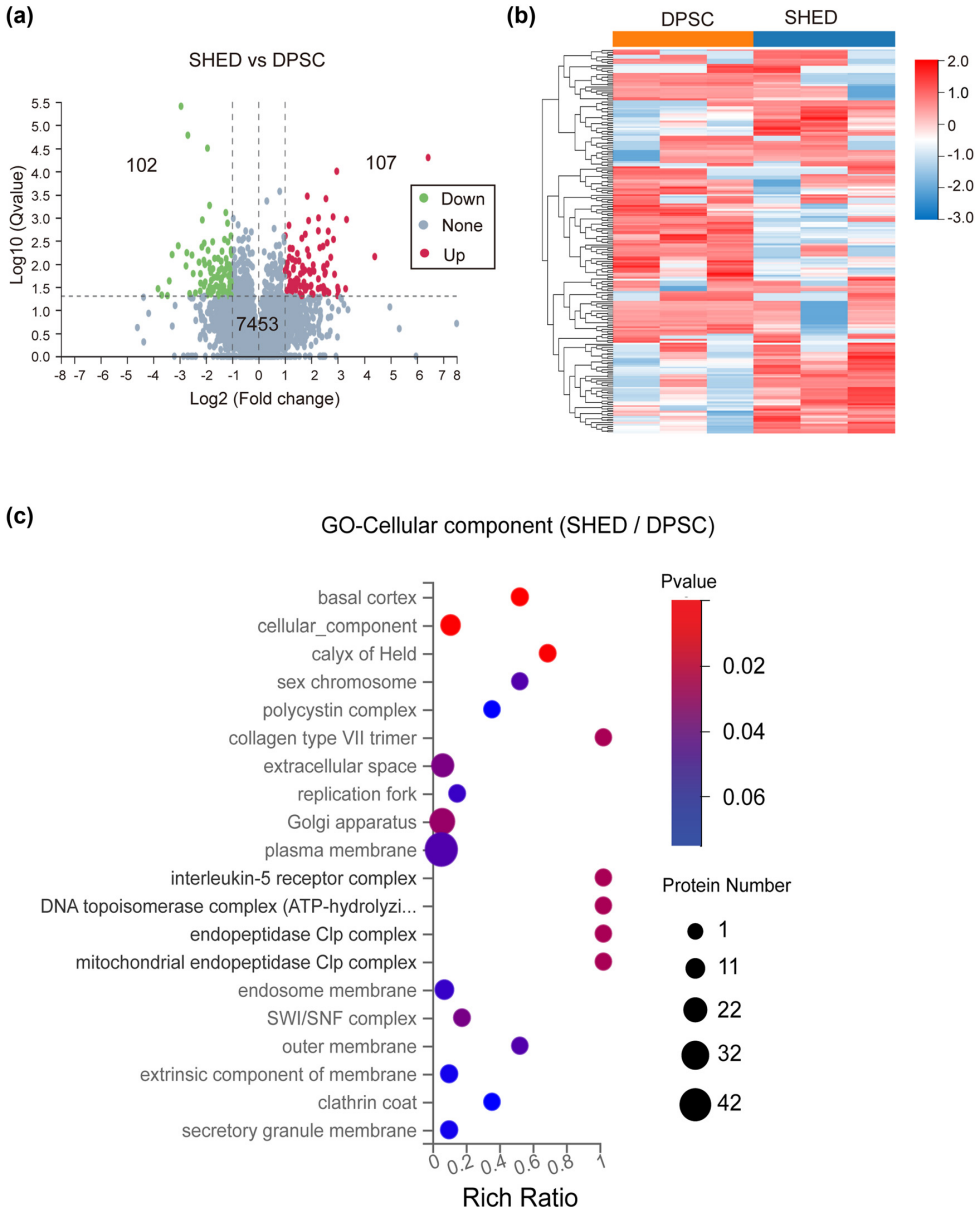
DEP analysis. (a) Volcano plot of the up- and down-regulated proteins between SHED and DPSC. (b) Clustering heatmap of the DEPs. (c) GO analysis of the CC for the differential proteins.

Differential expression analysis identified 107 proteins upregulated and 102 proteins downregulated in SHED compared to DPSC, with 7,453 proteins showing no significant changes (Supplementary File 1 with 209 DEPs). The volcano plot in Figure 2a visually represents the DEPs between DPSC and SHED. Figure 2b depicts the clustering analysis of these DEPs.

GO analysis of the DEPs revealed that a significant portion (42 proteins) were localized to the plasma membrane, with a notable number also found in the Golgi apparatus (32 proteins) or the extracellular space (22 proteins). The remaining DEPs were localized to other cellular compartments (Figure 2c).

### 3.3 KEGG classification and pathway enrichment highlights metabolic differences

**Figure 3:**
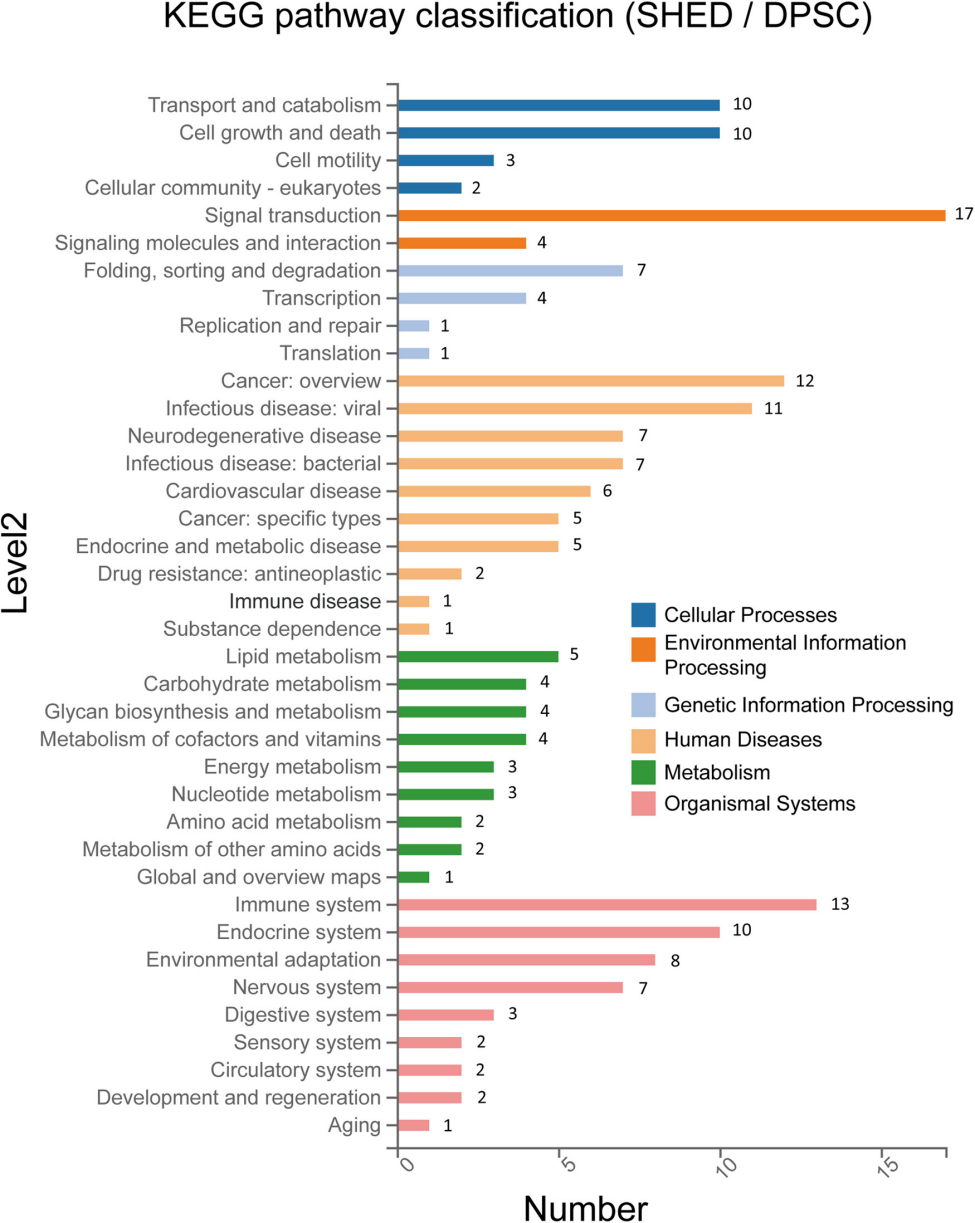
KEGG pathway classification of the differential proteins between SHED and DPSC. The *X*-axis represents the numbers of proteins associated with each pathway, while the *Y*-axis displays the enriched pathway names.

The DEPs were subjected to KEGG pathway classification, revealing significant enrichment across diverse functional categories (Figure 3). Notably, a substantial number of proteins (57) were associated with human diseases, indicating potential implications for cellular dysfunction and disease pathogenesis. Further investigation revealed a prominent role of metabolism, with 28 proteins enriched in metabolic pathways.

**Figure 4:**
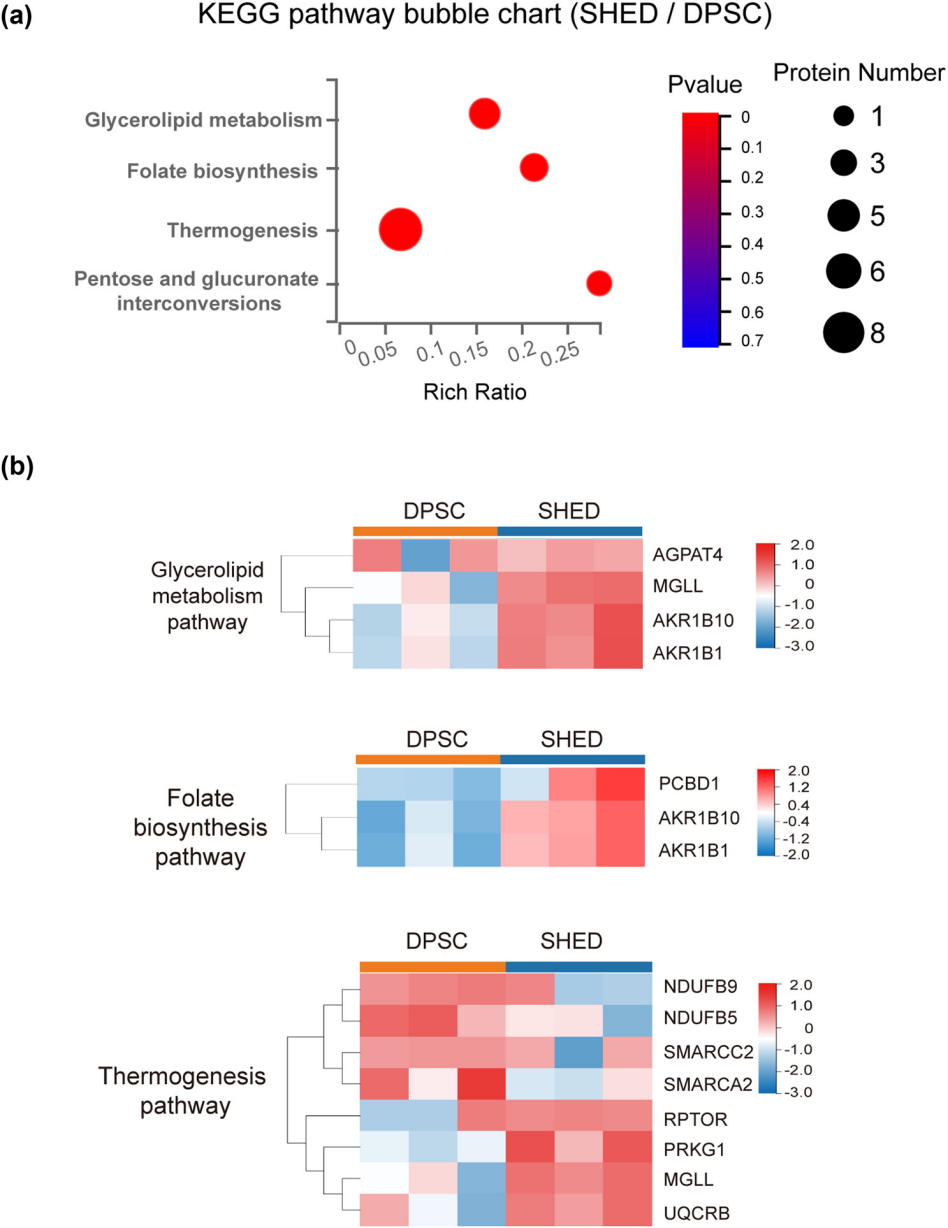
Bubble chart of KEGG pathway enrichment and clustering of representative pathways. (a) Bubble chart illustrating the significant KEGG pathway enrichment. (b) Clustering of the three most significantly enriched pathways.

Subsequent pathway enrichment analysis (Figure 4a) revealed distinct metabolic pathways that characterize the differences of DPSC and SHED. The bubble chart visualization highlighted the most significantly enriched pathways, with thermogenesis, glycerolipid metabolism, folate biosynthesis, and pentose/glucuronate interconversions emerging as key contributors to the observed metabolic divergence between these cell types.

Protein-pathway associations were as follows: eight proteins enriched in thermogenesis pathway, including NDUFB9, NDUFB5, SMARCC2, SMARCA2, RPTOR, PRKG1, MGLL, and UQCRB. Four proteins, AGPAT4, MGLL, AKR1B10, and AKR1B1, exhibited enrichment in glycerolipid metabolism pathway. Folate biosynthesis pathway encompassed three proteins: AKR1B10, AKR1B1, and PCBD1. AKR1B10 and AKR1B1 were also involved in pentose/glucuronate interconversions (Figure 4b).

Table 1 provides a detailed description of these proteins, highlighting differential expression patterns. Eight proteins (NDUFB family, SMARC, RPTOR, etc.) showed elevated expression in DPSC, while six proteins (AKR1B family, PRKG1, MGLL, etc.) were upregulated in SHED.

**Table 1:** List of the protein names and descriptions of the KEGG differential proteins

Protein name	Description	Ratio (SHED/DPSC)
NDUFB9	NADH dehydrogenase [ubiquinone] 1 beta subcomplex subunit 9	0.300
NDUFB5	NADH dehydrogenase [ubiquinone] 1 beta subcomplex subunit 5	0.474
SMARCC2	SWI/SNF related, matrix associated, actin dependent regulator of chromatin subfamily c member 2	0.371
SMARCA2	SWI/SNF related, matrix associated, actin dependent regulator of chromatin subfamily a member 2	0.494
RPTOR	Regulatory-associated protein of mTOR	0.350
AGPAT4	1-Acylglycerol-3-phosphate acyltransferase 4	0.162
TLR3	Toll-like receptor 3	0.304
SRRM2	Serine/arginine repetitive matrix protein 2	0.12
PRKG1	Protein kinase cGMP-dependent 1	2.662
MGLL	Monoglyceride lipase	2.183
UQCRB	Ubiquinol-cytochrome c reductase binding protein	2.088
AKR1B10	Aldo-keto reductase family 1 member B10	5.790
AKR1B1	Aldo-keto reductase family 1 member B1	6.653
PCBD1	Pterin-4-alpha-carbinolamine dehydratase 1	2.271

### 3.4 PPI network analysis unveils key regulatory nodes

**Figure 5:**
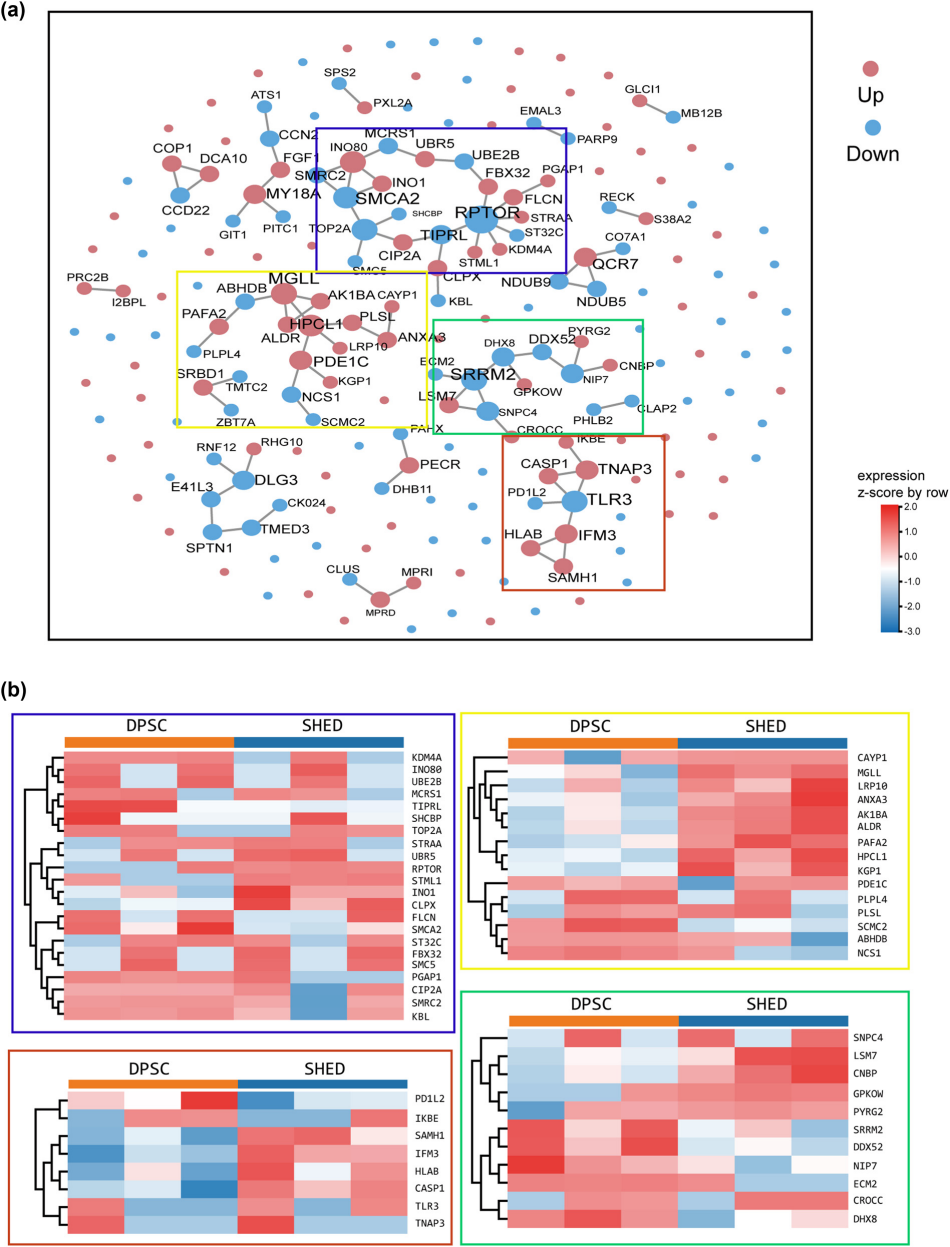
PPI network of the differential proteins. (a) PPI network of the differential proteins between DPSC and SHED. Red nodes represent upregulated proteins, while blue nodes represent downregulated proteins. The size of each nodes reflects the degree of relationship density. (b) Clustering heatmap depicting the four most significant protein interaction networks.

The PPI network analysis (Figure 5) revealed a complex interplay of protein interactions, highlighting four distinct modules enriched in specific pathways and proteins. These modules converged around four central proteins – RPTOR, MGLL, SRRM2, and TLR3 – acting as pivotal regulatory nodes within the network. RPTOR, with its extensive connections to seven proteins or pathways, emerges as a key player in stem cell functions. Its involvement in the mTOR-related autophagy pathway warrants particular attention. MGLL, linked to four proteins or pathways, participates in crucial metabolic processes, including thermogenesis and glycerolipid metabolism. Further investigation into the roles and interactions of these proteins within the PPI network is essential to comprehensively understand their contributions to the observed metabolic differences between DPSC and SHED.

## 4 Discussion

Proteomics, a fundamental technique for protein screening and functional analysis, has attained significant prominence within the scientific community. DIA mass spectrometry offers a distinct advantage over DDA by systematically fragmenting all detectable ions within a wide *m*/*z* range, regardless of their intensity. This comprehensive approach results in a wider dynamic range, enhanced reproducibility, improved sensitivity, greater accuracy in quantification, and potentially increased proteome coverage [18]. DIA has proven to be a widely adopted methodology in quantitative proteomic studies, particularly in plasma/serum proteomics, facilitating the identification of potential biomarkers across a diverse spectrum of diseases such as in 3P medicine (predictive, preventive, and personalized), cancer, etc. [19,20].

This study presents the first application of DIA proteomic technology to elucidate the proteomic landscape of DPSC and SHED. The comprehensive analysis identified over 7,000 proteins and 209 DEPs between DPSC and SHED, revealing distinct metabolic profiles between these two stem cell types. KEGG pathway and PPI network analysis revealed significant enrichment of proteins involved in metabolic pathways, particularly thermogenesis, glycerolipid metabolism, and folate biosynthesis. Notably, we identified the NDUFB protein family, SMARC family, RPTOR, and TLR3 proteins as prominent proteins in DPSC, while the AKR1B protein family, MGLL, and UQCRB were significantly enriched in SHED. Furthermore, RPTOR and MGLL emerged as central nodes in the PPI network, indicating their critical roles in cellular function. Importantly, these proteins were not previously reported in other dental stem cell proteomic studies.

Previous research on dental-related stem cells, such as DPSC, PDLSC, and SCAP, primarily relied on traditional two-dimensional gel electrophoresis proteomics. These studies primarily identified high-abundance proteins such as cytoskeletal proteins and cell adhesion molecules [21,22]. While subsequent gel-free high-throughput proteomic methods, like iTRAQ, tandem mass tags (TMT), and other shotgun approaches, have significantly expanded the repertoire of identified proteins and their associated functions and pathways, these studies mainly focused on comparisons between DPSC and other stem cells, such as PDLSC, SCAP, etc. [23–25], leaving the differential proteomic landscape between DPSC and SHED largely unexplored. A study investigating odontogenic differentiation from human DPSC using TMT-based proteomic analysis [23] identified 223 DEPs involved in cellular processes, metabolic processes, and biological regulation, and the protein expression levels of FBN1 and TGF-β2 validated by WB were consistent with the proteomic analysis. Lei et al. [24,25] employed TMT proteomics to compare the protein profiles of DPSC with those of PDLSC/SCAP. They identified high-scoring sub-networks in DPSC enriched with neural-related molecules, encoding cell vesicle transport and mitochondrial energy transfer to regulate cell proliferation and secretion factors. Additionally, a significant number of cell adhesion molecules were identified among the highly expressed molecules in PDLSC, indicating their potential role in providing cell attachment functions. While SCAP was characterized by a high expression of Niemann-Pick C1, a protein implicated in immune stability of SCAP.

Regarding the study between DPSC and SHED, Zainol Abidin et al. conducted a label-free quantitative proteomic analysis to investigate the differential expression of osteoblast-related proteins during osteogenic differentiation in DPSC and SHED. Their findings revealed significant differences in the expression of three proteins (PSMD11/RPN11, PLS3, and CLIC1) in stem cells from SHED, while a single protein (SYNCRIP) exhibited differential expression in DPSC [26]. However, a comprehensive proteomic analysis of undifferentiated DPSC and SHED has remained elusive until now. This research addressed this significant gap in the current area.

In the DEPs, DPSC exhibited significantly higher expression of the NDUFB (NADH dehydrogenase) family proteins, essential components of the mitochondrial electron transport chain responsible for energy production. This observation aligns with previous studies indicating the up-regulation of NDUF-related proteins during the differentiation of human GMSCs into neuron-like cells, suggesting a potential role for NDUFB in cell differentiation and metabolic reprogramming [27]. Furthermore, DPSC demonstrated elevated expression of RPTOR, a crucial component of the rapamycin target complex 1 (mTORC1), which plays a central role in the mTOR signaling pathway and governs essential cellular processes including body development, differentiation, and cell homeostasis [28]. Studies in mice have implicated RPTOR in maintaining the spermatogonial stem cell pool [29] and its deletion in hematopoietic stem/progenitor cells has been shown to lead to self-destructive innate immunity [30]. The increased RPTOR expression in DPSC suggests a higher autophagy capacity and enhanced regulation of cellular processes through the mTOR pathway.

Conversely, SHED displayed significantly higher expression of the AKR1B protein family, known for catalyzing the reduction of various aldehydes, including the aldehyde form of glucose [31]. This role in glucose metabolism is particularly relevant in diabetic complications, where AKR1B reduces glucose to sorbitol. Studies have shown that AKR1B1 expression increases during the differentiation of human multipotent adipose-derived stem cells, paralleling PGF2α release, while PGF2α receptor levels decline in early differentiation [32]. The elevated expression of AKR1B in SHED suggests a potential association with enhanced adipogenic differentiation ability. Other prominent proteins up-regulated in SHED include MGLL and UQCRB. MGLL, an enzyme responsible for hydrolyzing the endocannabinoid 2-arachidonoylglycerol, plays a crucial role in various physiological processes, including pain and nociperception [33]. UQCRB, a component of the mitochondrial respiratory chain, has been shown to play a pivotal role in hypoxic signaling-induced epithelial–mesenchymal transition (EMT), potentially serving as a therapeutic target for reversing EMT by inhibiting mitochondrial ROS production [34]. Additionally, N-acetyltransferase 10 has been demonstrated to repress Uqcr11 and Uqcrb, promoting heart regeneration by regulating their expression in mouse and human cardiomyocytes [35], further emphasizing the importance of UQCRB in metabolic reprogramming.

In conclusion, this study successfully established a DIA proteomics approach to analyze dental stem cells derived from permanent and deciduous teeth, leading to the identification of DEPs and enrichment of KEGG pathways. The findings revealed distinct metabolic profiles and key regulatory nodes between DPSC and SHED. Specifically, DPSC displayed higher expression of proteins belonging to the NDUFB family, SMARC family, RPTOR, and TLR3, implicated in pathways related to mitochondrial energy metabolism, mTOR-related autophagy, and immune signaling. In contrast, SHED exhibited higher expression of the AKR1B family, MGLL, and UQCRB, suggesting a stronger capacity for adipogenic differentiation and involvement in glycerolipid metabolism and thermogenesis pathways.

The identification of key regulatory proteins such as NDUFB, RPTOR, AKR1B, and MGLL provides valuable insights into the underlying mechanisms governing the differences between DPSC and SHED, and opens avenues for further investigation into their roles in stem cell function and potential therapeutic applications. However, it is important to acknowledge the limitations inherent in the use of a limited sample size and the absence of validation. Further research with larger sample sizes is necessary to validate these findings and confirm the observed differences.

## Supplementary Material

Supplementary material
