# Supplementary material for: Proteome differences of dental stem cells between permanent and deciduous teeth by data-independent acquisition proteomics
Source: Open Life Sci. 2025 Jan 29;20(1):20220998. doi: 10.1515/biol-2022-0998 (PMC11780257; doi:10.1515/biol-2022-0998)
Supplement: Supplementary material [file biol-2022-0998-sm.pdf]

Supplementary material

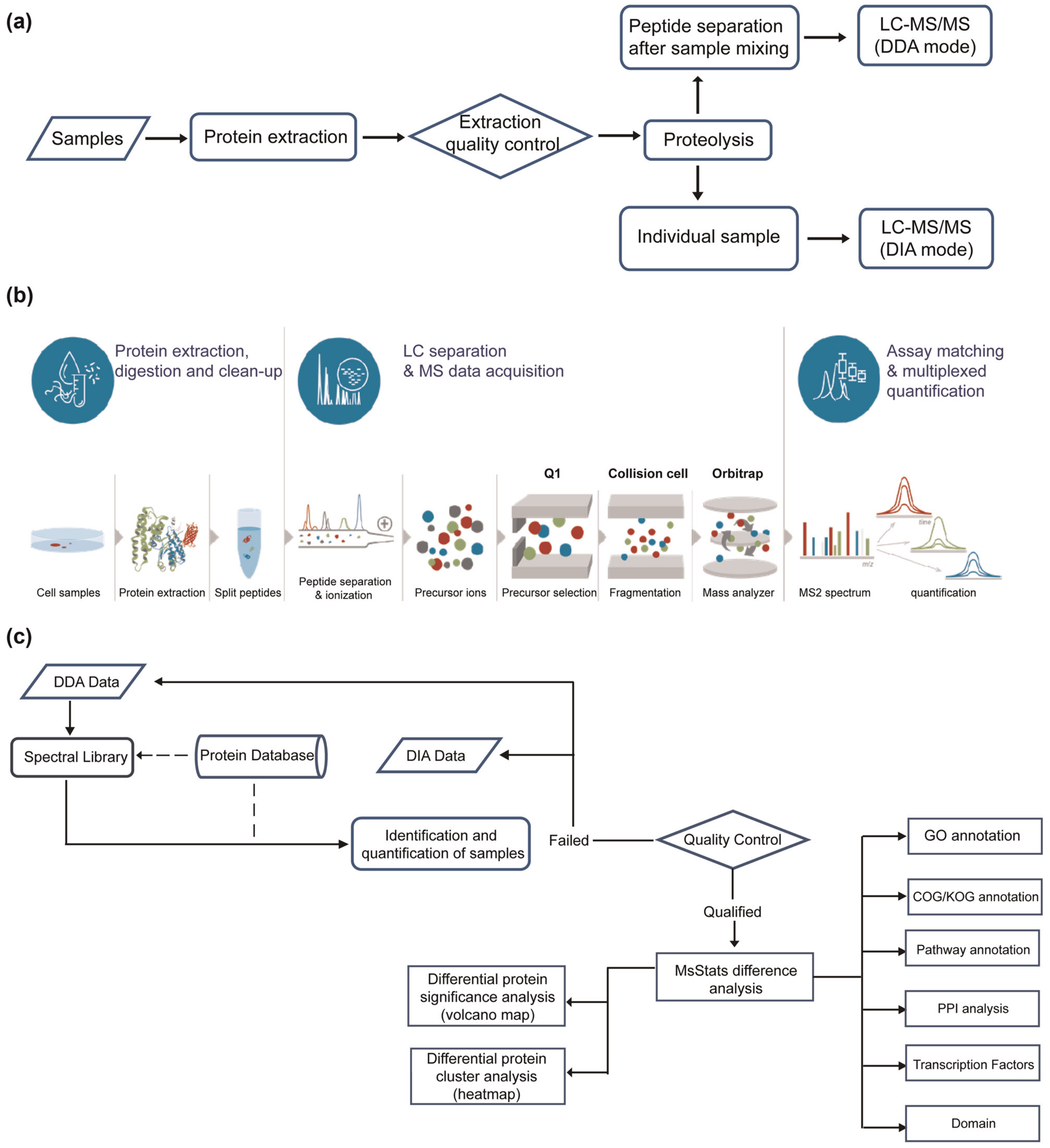

Figure S1: Workflow of DIA proteomics. (a) Schematic diagram of experimental procedures from cell samples to proteins and DIA. (b) Cartoon diagram of DIA workflow. (c) Process and content of DIA data analysis.

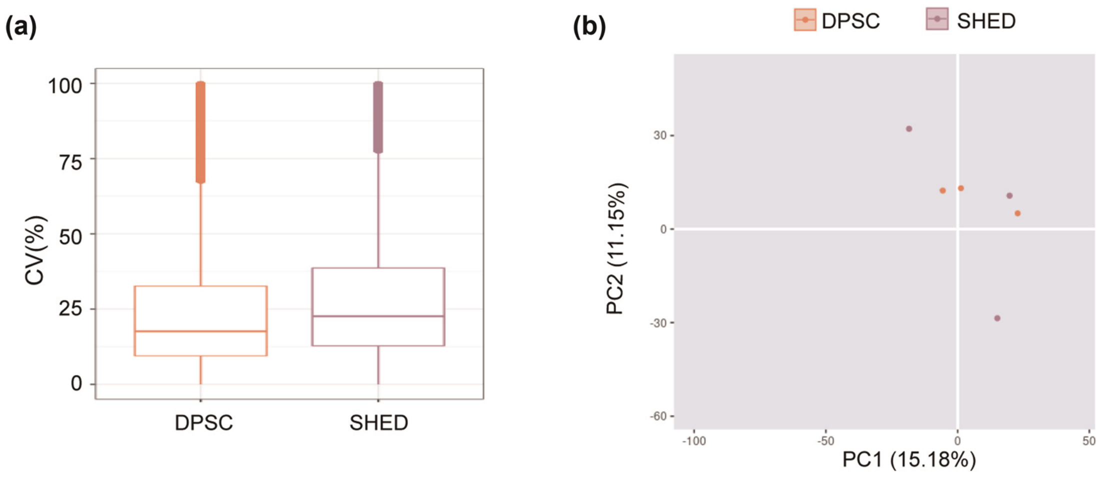

Figure S2: Quality control of DIA results. (a) CV distribution of the samples, the CV value was represented as Median ± 25%/4. (b) Principal component analysis (PCA), *X*-axis is the first principal component, *Y*-axis is the second principal component.

Table S1: Up regulated proteins in SHED vs DPSC

No	Protein ID	Symbol	Fold change (SHED/DPSC)	Description
1	sp|P26447|S10A4	S100A4	86.18	Protein S100-A4
2	sp|Q14956|GPNMB	GPNMB	21.15	Transmembrane glycoprotein NMB
3	sp|Q7Z5R6|AB1IP	APBB1IP	10.07	Amyloid beta A4 precursor protein-binding family B member 1-interacting protein
4	sp|Q9UHQ7|TCAL9	TCEAL9	9.83	Transcription elongation factor A protein-like 9
5	sp|Q5JSZ5|PRC2B	PRRC2B	8.10	Protein PRRC2B
6	sp|P13796|PLSL	LCP1	7.97	Plastin-2
7	sp|Q9ULG1|INO80	INO80	7.95	Chromatin-remodeling ATPase INO80
8	sp|Q92614|MY18A	MYO18A	7.79	Unconventional myosin-XVIIIa
9	sp|P46020|KPB1	PHKA1	7.35	Phosphorylase b kinase regulatory subunit alpha, skeletal muscle isoform
10	sp|Q9NR34|MA1C1	MAN1C1	7.15	Mannosyl-oligosaccharide 1,2-alpha-mannosidase IC
11	sp|Q8TCG1|CIP2A	CIP2A	7.07	Protein CIP2A
12	sp|P15121|ALDR	AKR1B1	6.65	Aldo-keto reductase family 1 member B1
13	sp|P13747|HLAE	HLA-E	6.33	HLA class I histocompatibility antigen, alpha chain E
14	sp|Q99973|TEP1	TEP1	6.22	Telomerase protein component 1
15	sp|P21580|TNAP3	TNFAIP3	6.07	Tumor necrosis factor alpha-induced protein 3
16	sp|O95639|CPSF4	CPSF4	6.06	Cleavage and polyadenylation specificity factor subunit 4
17	sp|Q92754|AP2C	TFAP2C	6.05	Transcription factor AP-2 gamma
18	sp|Q969G5|CAVN3	CAVIN3	5.86	Caveolae-associated protein 3
19	sp|O60218|AK1BA	AKR1B10	5.79	Aldo-keto reductase family 1 member B10
20	sp|Q9UBL6|CPNE7	CPNE7	5.69	Copine-7
21	sp|P01889|HLAB	HLA-B	5.64	HLA class I histocompatibility antigen, B alpha chain
22	sp|Q92585|MAML1	MAML1	5.58	Mastermind-like protein 1
23	sp|Q75T13|PGAP1	PGAP1	5.57	GPI inositol-deacylase
24	sp|Q9BRX8|PXL2A	PRXL2A	5.52	Peroxiredoxin-like 2A
25	sp|Q709C8|VP13C	VPS13C	5.51	Vacuolar protein sorting-associated protein 13C
26	sp|O95071|UBR5	UBR5	5.47	E3 ubiquitin-protein ligase UBR5
27	sp|Q15633|TRBP2	TARBP2	5.43	RISC-loading complex subunit TARBP2
28	sp|Q5TZA2|CROCC	CROCC	5.10	Rootletin
29	sp|Q96KB5|TOPK	PBK	4.99	Lymphokine-activated killer T-cell-originated protein kinase
30	sp|Q9UBI4|STML1	STOML1	4.82	Stomatin-like protein 1
31	sp|Q9Y5U2|TSSC4	TSSC4	4.81	Protein TSSC4
32	sp|Q9Y6J9|TAF6L	TAF6L	4.79	TAF6-like RNA polymerase II p300/CBP-associated factor-associated factor 65 kDa subunit 6L
33	sp|Q9H5V8|CDCP1	CDCP1	4.78	CUB domain-containing protein 1
34	sp|Q9Y3Z3|SAMH1	SAMHD1	4.28	Deoxynucleoside triphosphate triphosphohydrolase SAMHD1
35	sp|P05120|PAI2	SERPINB2	4.19	Plasminogen activator inhibitor 2
36	sp|Q92917|GPKOW	GPKOW	4.17	G-patch domain and KOW motifs-containing protein
37	sp|Q9H4I2|ZHX3	ZHX3	4.04	Zinc fingers and homeoboxes protein 3

(Continued)

Table S1: *Continued*

No	Protein ID	Symbol	Fold change (SHED/DPSC)	Description
38	sp|Q8IZ73|RUSD2	RPUSD2	4.03	RNA pseudouridylate synthase domain-containing protein 2
39	sp|Q14123|PDE1C	PDE1C	3.97	Calcium/calmodulin-dependent 3',5'-cyclic nucleotide phosphodiesterase 1C
40	sp|P15144|AMPN	ANPEP	3.92	Aminopeptidase N
41	sp|O00221|IKBE	NFKBIE	3.87	NF-kappa-B inhibitor epsilon
42	sp|Q8NFG4|FLCN	FLCN	3.75	Folliculin
43	sp|Q8NHY2|COP1	COP1	3.73	E3 ubiquitin-protein ligase COP1
44	sp|Q86VQ1|GLCI1	GLCCI1	3.71	Glucocorticoid-induced transcript 1 protein
45	sp|P20645|MPRD	M6PR	3.60	Cation-dependent mannose-6-phosphate receptor
46	sp|P37235|HPCL1	HPCAL1	3.59	Hippocalcin-like protein 1
47	sp|Q13938|CAYP1	CAPS	3.56	Calcyphosin
48	sp|Q5W0V3|FHI2A	FHIP2A	3.54	FHF complex subunit HOOK interacting protein 2A
49	sp|Q5K4L6|S27A3	SLC27A3	3.44	Solute carrier family 27 member 3
50	sp|P14317|HCLS1	HCLS1	3.41	Hematopoietic lineage cell-specific protein
51	sp|O94851|MICA2	MICAL2	3.40	[F-actin]-monooxygenase MICAL2
52	sp|P55042|RAD	RRAD	3.37	GTP-binding protein RAD
53	sp|Q9HAB3|S52A2	SLC52A2	3.30	Solute carrier family 52, riboflavin transporter, member 2
54	sp|Q9BY49|PECR	PECR	3.29	Peroxisomal trans-2-enoyl-CoA reductase
55	sp|Q01628|IFM3	IFITM3	3.21	Interferon-induced transmembrane protein 3
56	sp|Q7RTN6|STRAA	STRADA	3.17	STE20-related kinase adapter protein alpha
57	sp|Q5T7V8|GORAB	GORAB	3.17	RAB6-interacting golgin
58	sp|O14562|UBFD1	UBFD1	3.14	Ubiquitin domain-containing protein UBFD1
59	sp|Q8N5C6|SRBD1	SRBD1	3.11	S1 RNA-binding domain-containing protein 1
60	sp|Q8NBI5|S43A3	SLC43A3	3.10	Equilibrative nucleobase transporter 1
61	sp|P36941|TNR3	LTBR	3.00	Tumor necrosis factor receptor superfamily member 3
62	sp|Q9NPH2|INO1	ISYNA1	2.99	Inositol-3-phosphate synthase 1
63	sp|O00560|SDCB1	SDCBP	2.97	Syntenin-1
64	sp|Q96QD8|S38A2	SLC38A2	2.94	Sodium-coupled neutral amino acid transporter 2
65	sp|Q14596|NBR1	NBR1	2.88	Next to BRCA1 gene 1 protein
66	sp|P12429|ANXA3	ANXA3	2.82	Annexin A3
67	sp|Q9H1B7|I2BPL	IRF2BPL	2.77	Probable E3 ubiquitin-protein ligase IRF2BPL
68	sp|Q969P5|FBX32	FBXO32	2.76	F-box only protein 32
69	sp|A1A4S6|RHG10	ARHGAP10	2.73	Rho GTPase-activating protein 10
70	sp|Q9Y4C5|CHST2	CHST2	2.69	Carbohydrate sulfotransferase 2
71	sp|Q9NRF8|PYRG2	CTPS2	2.67	CTP synthase 2
72	sp|Q13976|KGP1	PRKG1	2.66	cGMP-dependent protein kinase 1
73	sp|Q969V6|MRTFA	MRTFA	2.64	Myocardin-related transcription factor A
74	sp|Q7Z5Q1|CPEB2	CPEB2	2.62	Cytoplasmic polyadenylation element-binding protein 2
75	sp|Q9H9S5|FKRP	FKRP	2.62	Ribitol 5-phosphate transferase FKRP

(Continued)

Table S1: *Continued*

No	Protein ID	Symbol	Fold change (SHED/DPSC)	Description
76	sp|Q6UX72|B3GN9	B3GNT9	2.54	UDP-GlcNAc:betaGal beta-1,3-N-acetylglucosaminyltransferase 9
77	sp|Q9BZL6|KPCD2	PRKD2	2.50	Serine/threonine-protein kinase D2
78	sp|O75164|KDM4A	KDM4A	2.49	Lysine-specific demethylase 4A
79	sp|Q14435|GALT3		2.48	Polypeptide N-acetylgalactosaminyltransferase 3
80	sp|Q7Z4F1|LRP10	LRP10	2.45	Low-density lipoprotein receptor-related protein 10
81	sp|Q9Y6H5|SNCAP	SNCAIP	2.43	Synphilin-1
82	sp|P62633|CNBP	CNBP	2.43	CCHC-type zinc finger nucleic acid binding protein
83	sp|O15533|TPSN	TAPBP	2.43	Tapasin
84	sp|Q6AI12|ANR40	ANKRD40	2.42	Ankyrin repeat domain-containing protein 40
85	sp|Q00613|HSF1	HSF1	2.41	Heat shock factor protein 1
86	sp|Q92870|APBB2	APBB2	2.39	Amyloid beta precursor protein binding family B member 2
87	sp|Q9NZ45|CISD1	CISD1	2.37	CDGSH iron-sulfur domain-containing protein 1
88	sp|Q9Y6N5|SQOR		2.37	Sulfide:quinone oxidoreductase, mitochondrial
89	sp|Q5QP82|DCA10	DCAF10	2.34	DDB1- and CUL4-associated factor 10
90	sp|P05230|FGF1	FGF1	2.32	Fibroblast growth factor 1
91	sp|Q99487|PAFA2	PAFAH2	2.30	Platelet-activating factor acetylhydrolase 2, cytoplasmic
92	sp|O76031|CLPX	CLPX	2.29	ATP-dependent Clp protease ATP-binding subunit clpX-like, mitochondrial
93	sp|P61457|PHS	PCBD1	2.27	Pterin-4-alpha-carbinolamine dehydratase
94	sp|Q6ZUK4|TMM26	TMEM26	2.24	Transmembrane protein 26
95	sp|Q13277|STX3	STX3	2.23	Syntaxin-3
96	sp|Q8WXE0|CSKI2	CASKIN2	2.23	Caskin-2
97	sp|P29466|CASP1	CASP1	2.22	Caspase-1
98	sp|Q9UK45|LSM7	LSM7	2.19	U6 snRNA-associated Sm-like protein LSm7
99	sp|Q53HV7|SMUG1	SMUG1	2.19	Single-strand selective monofunctional uracil DNA glycosylase
100	sp|Q99685|MGLL	MGLL	2.18	Monoglyceride lipase
101	sp|Q9H0N5|PHS2		2.12	Pterin-4-alpha-carbinolamine dehydratase 2
102	sp|Q03518|TAP1	TAP1	2.10	Antigen peptide transporter 1
103	sp|P28838|AMPL		2.09	Cytosol aminopeptidase
104	sp|P14927|QCR7	UQCRB	2.09	Cytochrome b-c1 complex subunit 7
105	sp|O95081|AGFG2	AGFG2	2.07	Arf-GAP domain and FG repeat-containing protein 2
106	sp|P11717|MPRI	IGF2R	2.01	Cation-independent mannose-6-phosphate receptor
107	sp|P19525|E2AK2	EIF2AK2	2.00	Interferon-induced, double-stranded RNA-activated protein kinase

Table S2: Down regulated proteins in SHED vs DPSC

No	Protein ID	Symbol	Ratio (SHED/DPSC)	Fold change	Description
1	sp|Q32P44|EMAL3	EML3	0.07	14.15	Echinoderm microtubule-associated protein-like 3
2	sp|Q8IWU6|SULF1	SULF1	0.08	12.83	Extracellular sulfatase Sulf-1
3	sp|Q8NEM2|SHCBP	SHCBP1	0.09	11.02	SHC SH2 domain-binding protein 1
4	sp|Q8N4P3|MESH1	HDDC3	0.10	10.51	Guanosine-3',5'-bis(diphosphate) 3'-pyrophosphohydrolase MESH1
5	sp|O60826|CCD22	CCDC22	0.10	9.76	Coiled-coil domain-containing protein 22
6	sp|Q9UQ35|SRRM2	SRRM2	0.12	8.31	Serine/arginine repetitive matrix protein 2
7	sp|Q8N394|TMTC2	TMTC2	0.13	7.72	Protein O-mannosyl-transferase TMTC2
8	sp|Q9UHI8|ATS1	ADAMTS1	0.15	6.77	A disintegrin and metalloproteinase with thrombospondin motifs 1
9	sp|P14621|ACYP2	ACYP2	0.15	6.63	Acylphosphatase-2
10	sp|Q96CN9|GCC1	GCC1	0.15	6.45	GRIP and coiled-coil domain-containing protein 1
11	sp|Q9NRZ5|PLCD	AGPAT4	0.16	6.16	1-acyl-sn-glycerol-3-phosphate acyltransferase delta
12	sp|Q8N2Q7|NLGN1	NLGN1	0.17	5.78	Neuroligin-1
13	sp|P10909|CLUS	CLU	0.18	5.61	Clusterin
14	sp|P55285|CADH6	CDH6	0.19	5.29	Cadherin-6
15	sp|O95980|RECK	RECK	0.21	4.81	Reversion-inducing cysteine-rich protein with Kazal motifs
16	sp|Q99611|SPS2	SEPHS2	0.21	4.66	Selenide, water dikinase 2
17	sp|O95248|MTMR5	SBF1	0.22	4.63	Myotubularin-related protein 5
18	sp|Q9Y3A3|PHOCN	MOB4	0.22	4.62	MOB-like protein phocein
19	sp|Q9Y2J2|E41L3	EPB41L3	0.22	4.46	Band 4.1-like protein 3
20	sp|A2RUS2|DEND3	DENND3	0.23	4.41	DENN domain-containing protein 3
21	sp|Q8TF42|UBS3B	UBASH3B	0.24	4.23	Ubiquitin-associated and SH3 domain-containing protein B
22	sp|Q99538|LGMN	LGMN	0.24	4.22	Legumain
23	sp|Q9HCJ1|ANKH	ANKH	0.24	4.20	Progressive ankylosis protein homolog
24	sp|Q9Y2D4|EXC6B	EXOC6B	0.24	4.12	Exocyst complex component 6B
25	sp|O75663|TIPRL	TIPRL	0.24	4.09	TIP41-like protein
26	sp|P41247|PLPL4	PNPLA4	0.26	3.85	Patatin-like phospholipase domain-containing protein 4
27	sp|Q8WUF8|F172A	FAM172A	0.26	3.84	Cotranscriptional regulator FAM172A
28	sp|Q7RTS9|DYM	DYM	0.26	3.79	Dymeclin
29	sp|Q6ZNW5|GDPP1	GDPGP1	0.26	3.78	GDP-D-glucose phosphorylase 1
30	sp|O95273|CCDB1	CCNDBP1	0.26	3.78	Cyclin-D1-binding protein 1
31	sp|Q96F05|CK024	C11orf24	0.27	3.66	Uncharacterized protein C11orf24
32	sp|Q8TCA0|LRC20	LRRC20	0.27	3.65	Leucine-rich repeat-containing protein 20
33	sp|O14832|PAHX	PHYH	0.28	3.62	Phytanoyl-CoA dioxygenase, peroxisomal
34	sp|Q9Y3Q3|TMED3	TMED3	0.28	3.60	Transmembrane emp24 domain-containing protein 3
35	sp|Q8N5W9|RFLB	RFLNB	0.28	3.53	Refilin-B
36	sp|Q86SQ0|PHLB2	PHLDB2	0.29	3.50	Pleckstrin homology-like domain family B member 2

(Continued)

Table S2: *Continued*

No	Protein ID	Symbol	Ratio (SHED/DPSC)	Fold change	Description
37	sp|P63146|UBE2B	UBE2B	0.29	3.49	Ubiquitin-conjugating enzyme E2 B
38	sp|Q13546|RIPK1	RIPK1	0.29	3.49	Receptor-interacting serine/threonine-protein kinase 1
39	sp|Q9BPU9|B9D2	B9D2	0.29	3.40	B9 domain-containing protein 2
40	sp|Q92629|SGCD	SGCD	0.30	3.36	Delta-sarcoglycan
41	sp|Q9Y6M9|NDUB9	NDUFB9	0.30	3.34	NADH dehydrogenase [ubiquinone] 1 beta subcomplex subunit 9
42	sp|O15455|TLR3	TLR3	0.30	3.29	Toll-like receptor 3
43	sp|Q9P2B2|FPRP	FPRP	0.31	3.27	Prostaglandin F2 receptor negative regulator
44	sp|P57768|SNX16	SNX16	0.31	3.25	Sorting nexin-16
45	sp|Q9Y2X7|GIT1	GIT1	0.31	3.19	ARF GTPase-activating protein GIT1
46	sp|Q9UHB7|AFF4	AFF4	0.33	3.07	AF4/FMR2 family member 4
47	sp|O75600|KBL	GCAT	0.33	2.99	2-amino-3-ketobutyrate coenzyme A ligase, mitochondrial
48	sp|Q6KCM7|SCMC2	SLC25A25	0.34	2.96	Calcium-binding mitochondrial carrier protein SCaMC-2
49	sp|Q96KR6|F210B	FAM210B	0.34	2.91	Protein FAM210B, mitochondrial
50	sp|O75113|N4BP1	N4BP1	0.34	2.90	NEDD4-binding protein 1
51	sp|Q96EZ8|MCRS1	MCRS1	0.35	2.88	Microspherule protein 1
52	sp|Q8N122|RPTOR	RPTOR	0.35	2.86	Regulatory-associated protein of mTOR
53	sp|Q15118|PDK1	PDK1	0.35	2.83	[Pyruvate dehydrogenase (acetyl-transferring)] kinase isozyme 1, mitochondrial
54	sp|O95365|ZBT7A	ZBTB7A	0.36	2.78	Zinc finger and BTB domain-containing protein 7A
55	sp|P11388|TOP2A	TOP2A	0.37	2.71	DNA topoisomerase 2-alpha
56	sp|Q9UH99|SUN2	SUN2	0.37	2.70	SUN domain-containing protein 2
57	sp|Q8TAQ2|SMRC2	SMARCC2	0.37	2.69	SWI/SNF complex subunit SMARCC2
58	sp|Q8NFV4|ABHDB	ABHD11	0.37	2.69	Protein ABHD11
59	sp|Q9Y221|NIP7	NIP7	0.38	2.66	60S ribosome subunit biogenesis protein NIP7 homolog
60	sp|Q9BY42|RTF2	RTF2	0.38	2.65	Replication termination factor 2
61	sp|Q9HC35|EMAL4	EML4	0.38	2.63	Echinoderm microtubule-associated protein-like 4
62	sp|P29279|CCN2	CCN2	0.38	2.60	CCN family member 2
63	sp|Q96BH1|RNF25	RNF25	0.38	2.60	E3 ubiquitin-protein ligase RNF25
64	sp|Q8IY18|SMC5	SMC5	0.39	2.60	Structural maintenance of chromosomes protein 5
65	sp|Q9UKF7|PITC1	PITPNC1	0.39	2.55	Cytoplasmic phosphatidylinositol transfer protein 1
66	sp|Q9H7P6|MB12B	MVB12B	0.40	2.52	Multivesicular body subunit 12B
67	sp|O75400|PR40A	PRPF40A	0.40	2.52	Pre-mRNA-processing factor 40 homolog A
68	sp|Q0VDI3|TM267	TMEM267	0.40	2.50	Transmembrane protein 267
69	sp|O75122|CLAP2	CLASP2	0.40	2.48	CLIP-associating protein 2
70	sp|Q96MG7|NSE3	NSMCE3	0.41	2.46	Non-structural maintenance of chromosomes element 3 homolog
71	sp|Q9Y2R4|DDX52	DDX52	0.41	2.46	Probable ATP-dependent RNA helicase DDX52
72	sp|Q86UX6|ST32C	STK32C	0.41	2.45	Serine/threonine-protein kinase 32C

(Continued)

Table S2: *Continued*

No	Protein ID	Symbol	Ratio (SHED/DPSC)	Fold change	Description
73	sp|Q9H867|MT21D	VCPKMT	0.41	2.43	Protein N-lysine methyltransferase METTL21D
74	sp|Q9GZN8|CT027	C20orf27	0.42	2.38	UPF0687 protein C20orf27
75	sp|Q8IXQ6|PARP9	PARP9	0.42	2.38	Protein mono-ADP-ribosyltransferase PARP9
76	sp|Q9ULF5|S39AA	SLC39A10	0.42	2.36	Zinc transporter ZIP10
77	sp|Q8TE77|SSH3	SSH3	0.43	2.32	Protein phosphatase Slingshot homolog 3
78	sp|Q96HY7|DHTK1	DHTKD1	0.43	2.30	Probable 2-oxoglutarate dehydrogenase E1 component DHKTD1, mitochondrial
79	sp|P62166|NCS1	NCS1	0.44	2.29	Neuronal calcium sensor 1
80	sp|Q14257|RCN2	RCN2	0.44	2.27	Reticulocalbin-2
81	sp|Q9NVW2|RNF12	RLIM	0.45	2.24	E3 ubiquitin-protein ligase RLIM
82	sp|P36955|PEDF	SERPINF1	0.45	2.24	Pigment epithelium-derived factor
83	sp|P43007|SATT	SATT	0.45	2.22	Neutral amino acid transporter A
84	sp|O00584|RNT2	RNASET2	0.45	2.22	Ribonuclease T2
85	sp|Q92536|YLAT2	SLC7A6	0.45	2.21	Y+L amino acid transporter 2
86	sp|Q92796|DLG3	DLG3	0.45	2.21	Disks large homolog 3
87	sp|Q9Y3E1|HDGR3	HDGFL3	0.46	2.17	Hepatoma-derived growth factor-related protein 3
88	sp|Q96RF0|SNX18	SNX18	0.46	2.16	Sorting nexin-18
89	sp|Q969Z0|FAKD4	FAKD4	0.46	2.16	FAST kinase domain-containing protein 4
90	sp|Q6UWJ1|TMCO3	TMCO3	0.46	2.16	Transmembrane and coiled-coil domain-containing protein 3
91	sp|Q02388|CO7A1	COL7A1	0.47	2.14	Collagen alpha-1(VII) chain
92	sp|Q5SXM2|SNPC4	SNAPC4	0.47	2.13	snRNA-activating protein complex subunit 4
93	sp|Q9Y646|CBPQ	CPQ	0.47	2.12	Carboxypeptidase Q
94	sp|O43674|NDUB5	NDUFB5	0.47	2.11	NADH dehydrogenase [ubiquinone] 1 beta subcomplex subunit 5, mitochondrial
95	sp|Q15648|MED1	MED1	0.48	2.08	Mediator of RNA polymerase II transcription subunit 1
96	sp|Q9BQ51|PD1L2	PDCD1LG2	0.48	2.07	Programmed cell death 1 ligand 2
97	sp|O94769|ECM2	ECM2	0.48	2.06	Extracellular matrix protein 2
98	sp|Q14562|DHX8	DHX8	0.48	2.06	ATP-dependent RNA helicase DHX8
99	sp|Q13813|SPTN1	SPTAN1	0.49	2.06	Spectrin alpha chain, non-erythrocytic 1
100	sp|Q8NDI1|EHBP1	EFBP1	0.49	2.06	EH domain-binding protein 1
101	sp|Q8NBQ5|DHB11	HSD17B11	0.49	2.03	Estradiol 17-beta-dehydrogenase 11
102	sp|P51531|SMCA2	SMARCA2	0.49	2.02	Probable global transcription activator SNF2L2
